# Perioperative hypotension: causes and remedies

**DOI:** 10.1186/s44158-022-00045-8

**Published:** 2022-04-14

**Authors:** Fabio Guarracino, Pietro Bertini

**Affiliations:** Department of Anaesthesia and Critical Care Medicine, Azienda Ospedaliero Universitaria Pisana, Via Paradisa 2, 56123 Pisa, Italy

**Keywords:** Anesthesia, Surgery, Perioperative, Arterial pressure, Hypotension, Treatment

## Abstract

**Background:**

Arterial hypotension is common in patients during surgery and those admitted to the intensive care unit (ICU) postoperatively. Perioperative arterial hypotension reportedly significantly affects surgical patients’ outcomes. Blood pressure level is the most crucial factor that influences organ perfusion. Hypoperfusion and organ dysfunction are correlated based on their severity associated with hypotension. As several factors can cause intraoperative hypotension, anesthetists must promptly identify the etiology for appropriate treatment and revert the patient’s hemodynamic profile.

**Objectives:**

This review discusses the concept of perioperative hypotension, identifies its effects in clinical situations, and provides remedies and techniques to predict and avoid its significant consequences.

**Conclusions:**

The primary determinant of organ perfusion is blood pressure. On the other hand, profound hypotension is common in surgical patients and is connected to hypoperfusion and organ failure. Currently, hypotension is addressed once low blood pressure levels are recorded. Early detection of oncoming hypotension or its clinical prediction is of paramount importance in allowing the clinician to treat hypotension and reduce the incidence and length of hypotensive episodes promptly and aggressively.

## Background

When examining organ-specific outflow pressure, mean arterial pressure (MAP) is the fundamental predictor of end-organ perfusion. Healthy people’s blood pressure changes greatly during the day, although it may be kept steady within certain parameters. Patients who are undergoing surgery, who are critically ill, and who have tissue hypoperfusion and organ damage are more likely to experience profound hypotension [1, 2].

Intraoperative hypotension has been linked to a higher risk of postoperative mortality [3, 4], myocardial injury after non-cardiac surgery (MINS) [5], myocardial infarction [6], cardiogenic shock [7], acute renal failure [8], delirium [2], and stroke [9] in patients undergoing non-cardiac surgery under general anesthesia [10–14]. Although blood flow autoregulation protects the brain, heart, and kidneys from hypotension-induced hypoperfusion, blood pressure is almost entirely responsible for perfusion of other organ systems, particularly splanchnic organs, such as the stomach, liver, and pancreas, which have a low blood flow autoregulation capacity [15]. Intermittent or continuous blood pressure monitoring using invasive or non-invasive measurement methods is the standard of care in perioperative and critical care medicine to preserve patient safety and improve perfusion pressure. To reduce the overall degree of severe hypotension, imminent hypotension is increasingly being recognized and treated early.

In the present review, we discuss the concept of hypotension, identify its effects in most clinical situations, and provide remedies and techniques to predict and avoid its significant consequences.

## The physiology of blood pressure

Systolic arterial pressure (SAP) is the maximum pressure measured in the arterial vasculature and arises from the contraction of the left ventricle. It depends on stroke volume (SV), heart rate (HR), systemic vascular resistance (SVR), the distensibility of the aorta, and the large arteries near the heart. The lowest pressure recorded in the arterial tree is called diastolic arterial pressure (DAP). DAP is influenced by SVR, compliance of the vasculature (“windkessel model” of the vasculature), the duration of diastole, and the duration of the cardiac cycle [16].

Mean arterial pressure (MAP) is the average arterial pressure throughout one cardiac cycle.

Cardiac output (CO) and SVR are the determinants of MAP. The MAP value is under the control of the renin-angiotensin-aldosterone system and the autonomic nervous system, whose complex physiology is well beyond the scope of this review. However, it is important to emphasize that anesthesiologists should be aware of the implications of changes in both peripheral resistance and circulating volume on MAP via regulation of the carotid sinus and aortic arch baroceptors and the renin-angiotensin-aldosterone cascade. In fact, most of the anesthesia drugs and techniques routinely used and many factors related to the surgical procedure occurring perioperatively, of which blood loss is a caricatural example, can alter either the vascular tone or the plasma volume.

The goal of physiological regulation of blood pressure and flow with the oxygen content of arterial blood is to facilitate adequate tissue oxygenation.

Systemic perfusion pressure (PP) regulates the blood flow of the whole organism [17]. PP is calculated as MAP–central venous pressure (CVP). When analyzing the single end-organ perfusion instead, it is less correct to refer to CVP as downstream pressure; hence, the closing pressure (CP) of the respective area or tissue should be taken into account.

The best example of such a concept is the perfusion of the brain, which, under physiological conditions, depends on the cerebral perfusion pressure (CPP), which is calculated as MAP–intracranial pressure (ICP), where ICP is assimilated CP or downstream pressure. An analog case occurs under pathological conditions in other body areas, such as in the muscle compartment or the abdomen, when compartment syndrome occurs.

In some organs, such as the brain, spinal cord, kidneys, and heart, blood flow is kept constant within MAP limits of autoregulation blood flow [18]. On the other hand, autoregulation occurs within a defined range of blood pressure. For example, for cerebral perfusion, MAP values in healthy individuals are 60–160 mmHg, within which blood flow is kept constant.

Kidneys blood flow is maximal within 70–130 mmHg of MAP values [19]; therefore, glomerular filtration is optimal in this pressure range.

Myocardial blood flow is autoregulated within certain limits of coronary perfusion pressure (CoPP), although myocardial perfusion is not maintained at a constant value; instead, it is adjusted to the current myocardial O_2_ demand [20].

In daily routine, blood pressure is often used as a surrogate for blood flow. Nevertheless, perioperative changes in blood pressure are unreliable substitutes for simultaneous changes in cardiac output (CO), as demonstrated in 402 anesthetized patients undergoing different surgical procedures [21]. In each case, the blood pressure parameters SAP, DAP, MAP, PP, and CO were measured before and after the administration of a fluid bolus of 500 ml colloidal solution for 10–20 min. As the CO increased by > 15%, a positive response to the volume expansion was evaluated. Although blood pressure parameters increased more in responders than in non-responders, relative changes in pressure values showed low sensitivity and specificity. Thus, the relative pressure changes failed to predict changes in CO in more than half of the patients. Similar results were found in patients with septic shock [19].

## Defining arterial hypotension

Whereas blood pressure management is a pillar of anesthetic care perioperatively, the definition of hypotension remains challenging. Although there are numerous references that support the need for perioperative blood pressure regulation [22–25], at present, there are no universally accepted perioperative blood pressure thresholds to define hypotension [26].

Weinber and colleagues published a comprehensive study of intraoperative hypotension criteria in adults who underwent non-cardiac surgery [27]. Hypotension was mostly defined by changes in SAP, MAP, or a combination of the two. The investigators tried to determine whether studies reported an absolute threshold value for hypotension, hypotension as a change from a pressure baseline value, a baseline blood pressure for reporting relative threshold values, and a methodology for determining the severity of hypotension.

**Fig. 1:**
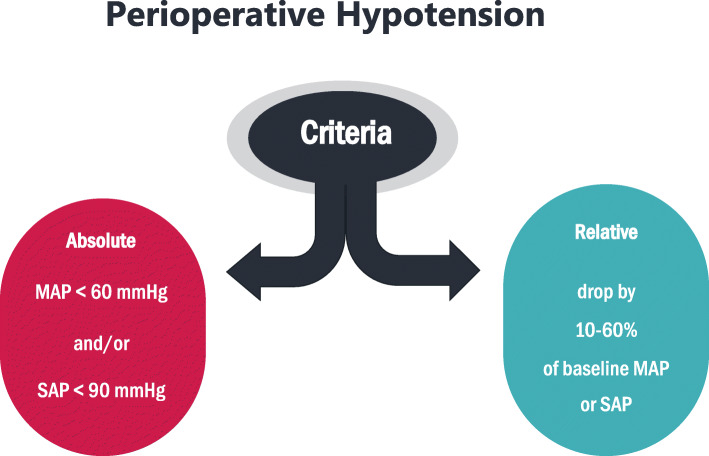
Definitions of perioperative hypotension

The majority of studies reported a MAP < 60 mmHg and/or a SAP < 90 mmHg as absolute numerical thresholds for hypotension. A total of 126 studies used a relative threshold to represent hypotension, most of which showed a percentage decrease (10 to 60%) from baseline for either MAP or SAP (Fig. 1). Other hypotension definitions refer to a blood pressure requiring therapeutic interventions, such as volume expansion or vasoactive medications.

## Causes of perioperative hypotension

The causes of perioperative hypotension can be related to the patient, anesthesia, or surgery.

**Fig. 2:**
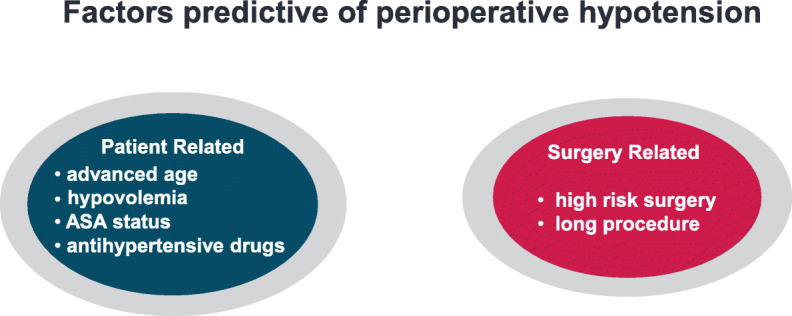
Predicting factors of perioperative hypotension

Preoperative risk factors associated with hypotension are advanced age, low blood pressure before anesthesia induction, hypovolemia [28–30], higher American Society of Anesthesiologists (ASA) status, chronic treatment with antihypertensive drugs, and planned high-risk surgery [31–34](Fig. 2).

Whereas beta blockers and calcium antagonists have direct recommendations relating to the perioperative regime, because they have been associated with both injury and benefit, there is controversy about whether angiotensin-converting enzyme inhibitors/angiotensin II receptor blockers (ACE-Is/ARBs) should be maintained in the perioperative period. Intraoperative hypotension caused by ACE-I and ARB medication that persists throughout the perioperative period [35] has been linked to severe perioperative morbidity [36] and has prompted some clinicians to discontinue treatment. The current perioperative guidelines are divided in their recommendations for continuing or stopping ACEIs/ARBs during surgery.

According to the 2014 American College of Cardiology/American Heart Association guidelines [31], continuing therapy before surgery is appropriate. Additionally, if therapy is stopped, it should be resumed as soon as clinically possible, whereas the Canadian Cardiovascular Society’s most recent guidelines [32] recommend skipping therapy 24 h before surgery (strong recommendation, low quality of evidence).

The European Society of Cardiology/European Society of Anaesthesiology approach [33], on the other hand, bases its recommendations on the indication for ACE-I/ARB treatment, including 24-h termination if the medication is indicated for hypertension and continuation if it is prescribed for heart failure or left ventricular systolic dysfunction [33].

In addition, if these latter patients are not on ACE-I/ARB medication prior to surgery, recommendations advise starting it one week before surgery [34].

Intraoperative hypotension has a complex origin, and it is most prevalent in patients undergoing surgery under general and neuraxial anesthesia [9, 37–39]. The main factors contributing to hypotension intraoperatively are excessive depth of anesthesia [40, 41], blood loss [42], and vasodilation.

Hypotension is also common in postoperative care in the ICU. Several causes can be listed, including myocardial ischemia [43], hypovolemia, arrhythmias [44, 45], vasoplegia, dynamic left ventricle outflow tract (LVOT) obstruction [46, 47], pneumothorax [48], tamponade [49], pulmonary embolism [50], sepsis [51], and bleeding [52].

## Remedies to treat hypotension

**Fig. 3:**
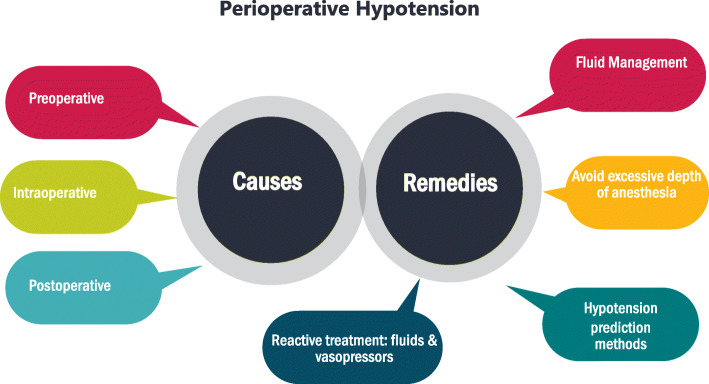
Causes and remedies of perioperative hypotension

Although patient-related causes of hypotension are not modifiable, there is room for the anesthetic team to intervene on anesthesia- and surgery-related causes to prevent or promptly reverse hypotension (Fig. 3, Table 1).

**Table 1:** Specific causes and remedies for perioperative hypotension

Causes	Remedy
**Preoperative**	
Hypovolemia	Replace volume depletion with fluids according to the current guidelines or local protocol, minimize starvation if possible [28–30].
ACE-Is/ARBs	Suspend medications in the perioperative period [31–34].
**Intraoperative**	
Excessive depth of anesthesia	Minimize excessive anesthesia intensity by monitoring the depth of the anesthetic plane [40, 41].
Neuraxial blockade	Administer intravenous fluids, ephedrine, phenylephrine, ondansetron, leg compression [39].
Blood loss	Replace volume depletion with fluids and blood products according to the current guidelines or local protocol [42].
Myocardial ischemia	Hemodynamic and biohumoral markers assessment [31], intraoperative TEE to detect and confirm alteration in myocardial contractility [43].
**Postoperative**	
Myocardial ischemia	Appropriate hemodynamic and biohumoral markers assessment [31].
Hypovolemia	Replace volume depletion with fluids and blood products according to the current guidelines or local protocol
Arrhythmias	Monitor ECG and correct arrythmia using ACLS, ALS or analogue protocols [31, 44, 45].
Dynamic LVOT Obstruction	Administer fluids, give medications to lower heart rate (e.g beta blockers), stop beta agonists [46, 47].
Pneumothorax	Treatment as needed (ranges from tight follow-up to chest tube insertion to thoracic surgery) [48].
Tamponade	Drainage of the pericardial space [49].
Pulmonary embolism	Treatment according to the guidelines or local protocols [50].
Sepsis	Treatment according to the Surviving Sepsis Guidelines [51].
Bleeding	Replace volume depletion, monitor coagulation and correct shortage of coagulation determinants if possible, according to the current protocols [42, 52]

*ACE-Is/ARBs* angiotensin converting enzyme inhibitors/angiotensin II receptor blockers, *ECG* electrocardiogram, *ACLS* advanced cardiac life support, *ALS* advanced life support

Treatment of any specific cause of hypotension should be pursued in a timely and appropriate manner. To do so, identifying and correcting the underlying pathophysiologic mechanisms, such as decreased cardiac preload, altered cardiac afterload, or reduced myocardial contractility, is pivotal for causal treatment of hypotension [12].

Predicting hypotensive episodes may lead to preventive treatment and assist in avoiding hypotension. Recently, a new technology based on AI has shown promise in predicting hypotension. Hatib et al. developed a “hypotension prediction index” (HPI) to predict real-time hypotension [53]. They used machine learning to analyze many hemodynamic variables collected from the arterial blood pressure waveform in real time. After the verified the method, the model predicted arterial hypotension 15 min ahead of time, with a sensitivity of 88% and a specificity of 87%.

Wijnberge et al. [54] presented the Hypotension Prediction (HYPE) trial, in which 68 patients undergoing elective non-cardiac surgery were randomly assigned to either an AI early warning system for intraoperative treatment (intervention group) or conventional care (control group). The goal of their study was to investigate whether the intervention decreased the depth and duration of intraoperative hypotension. As evidenced by the primary result of a decreased time-weighted average of intraoperative hypotension, the trial showed that the intervention successfully reduced patients' exposure to hypotension.

Although physicians may use arterial pulse pressure waveforms to make reasonable judgments about the risk of forthcoming episodes of hypotension, there is a good chance that an AI system might make more accurate predictions [55].

## Conclusions

Blood pressure is a critical factor in determining organ perfusion. Perioperative hypotension is common and is linked to hypoperfusion and organ failure. Therefore, optimal management of arterial blood pressure is required in the perioperative setting to avoid complications.

Currently, hypotension is addressed once low blood pressure levels are recorded. Preoperative risk stratification for perioperative hypotension, intraoperative, and postoperative early detection of oncoming hypotension or its clinical prediction allow the clinician to treat hypotension and reduce the incidence and length of hypotensive episodes. Machine learning-based algorithms have recently been applied to predict hypotension. However, clinical trials are needed to confirm the ability of new technologies to effectively predict hypotension cases.

Based on current knowledge, anesthesiologists should carefully screen patients at risk for hypotension at the time of the preoperative evaluation, pay a high level of attention to the perioperative clinical management to avoid hypotensive episodes, and treat the disease aggressively in a timely fashion.

## Data Availability

Not applicable

## Abbreviations

ICU: Intensive care unit
MAP: Mean arterial pressure
SAP: Systolic arterial pressure
DAP: Diastolic arterial pressure
SVR: Systemic vascular resistances
CVP: Central venous pressure
CPP: Cerebral perfusion pressure
ICP: Intracranial pressure
CO: Cardiac output
PP: Perfusion pressure
ASA: American Society of Anesthesiologists status
AI: Artificial intelligence
HPI: Hypotension prediction index
